# Stevens‐Johnson syndrome/toxic epidermal necrolysis predictive models are not effective when applied by non‐dermatologists: A single‐institution prospective study

**DOI:** 10.1002/ski2.292

**Published:** 2023-09-12

**Authors:** Hunter J. Pyle, Arturo R. Dominguez, Melissa M. Mauskar, Cristina Thomas

**Affiliations:** ^1^ Department of Dermatology University of Texas Southwestern Medical Center Dallas Texas USA; ^2^ Department of Internal Medicine University of Texas Southwestern Medical Center Dallas Texas USA; ^3^ Department of Obstetrics and Gynecology University of Texas Southwestern Medical Center Dallas Texas USA

Dear Editor,

Stevens‐Johnson syndrome and toxic epidermal necrolysis (SJS/TEN) are a spectrum of life‐threatening mucocutaneous diseases with high mortality. Early transfer to a burn centre may reduce patient mortality.^1^ Lack of standardized triage guidelines and access to dermatologist evaluation contribute to inappropriate transfer of patients who do not have SJS/TEN and do not require burn unit care.^2^, ^3^ Two predictive models to aid in differentiation of SJS/TEN from clinical mimickers have been developed and externally validated with good discriminative ability.^4^, ^5^ These models were developed and validated based on physical examination data from experienced inpatient dermatologists. Here, we sought to assess the performance of these models in the context of patient assessments by non‐dermatologists.

A multidisciplinary telemedicine system to triage SJS/TEN transfer requests was implemented with institutional review board exemption as a quality improvement study at Parkland Memorial Hospital. Centres requesting transfer completed a standardized questionnaire and provided photos for inpatient dermatologist and burn team review. Results from March 2022 to January 2023 were analysed. In each case, the probability of SJS/TEN was calculated using the predictive models. Model variables included presence of Nikolsky sign, atypical targets, fever, and either mucosal involvement or lymphopenia.^4^ Model discriminatory abilities were quantified using area under the receiver operating characteristics curve (AUROC) and model performance was assessed using sensitivity, specificity, positive predictive value (PPV), and negative predictive value (NPV). A cut‐off value of 0.40 was previously reported to hold high absolute accuracy in this model and was applied here to assess performance metrics.^4^ Initial inpatient dermatologist triage impressions of photographs and questionnaires were used as criterion standard.^6^ Standard descriptive statistics were presented as absolute and relative frequencies for categorical variables.

In total, 55 transfer requests were reviewed, and 47 requests were completed with sufficient information to employ either predictive model. Background characteristics and summary questionnaire results are displayed in Table 1. Upon initial review of photographs and questionnaire by the inpatient dermatology team, 13 patients were considered to have presentations suspicious for SJS/TEN while 34 patients had presentations consistent with a non‐SJS/TEN entity. When mucosal involvement was included in the model, the AUROC was 0.6290 (95% confidence interval, 0.44–0.82; *p* = 0.18). With a cut‐off value of 0.40, the model yielded a 61.5% sensitivity, 44.1% specificity, 29.6% PPV, and 75.0% NPV. When lymphopenia was included in the model, the AUROC was 0.5925 (95% confidence interval, 0.39–0.79; *p* = 0.37).

**TABLE 1:** Background characteristics and questionnaire results from non‐dermatologists of patients with Stevens‐Johnson syndrome and toxic epidermal necrolysis compared to non‐Stevens‐Johnson syndrome and toxic epidermal necrolysis based on inpatient dermatologist triage impression.

	Inpatient dermatologist triage impression	
Background characteristics and questionnaire responses	SJS/TEN (*n* = 13), *n* (%)	Non‐SJS/TEN (*n* = 34), *n* (%)
Demographics		
Sex		
Female	5/13 (38.5%)	20/34 (58.8%)
Male	8/13 (61.5%)	14/34 (41.2%)
Age, median (IQR), *y*	57 (46)	59 (33)
Questionnaire		
Nikolsky positive	7/13 (53.8%)	14/34 (41.2%)
Atypical targets	4/13 (30.8%)	14/34 (41.2%)
Skin pain	12/12 (100.0%)	29/34 (85.3%)
Mucosal involvement	12/13 (92.3%)	25/34 (73.5%)
Fever >100.4°F	8/13 (61.5%)	3/34 (8.8%)
Lymphopenia	4/11 (36.4%)	7/29 (24.1%)
Suspicious drug	13/13 (100.0%)	25/34 (73.5%)

Abbreviations: IQR, interquartile range; SJS/TEN, Stevens‐Johnson syndrome/toxic epidermal necrolysis.

Results here suggest that these models have poor SJS/TEN predictive performance when applied to data collected by non‐dermatologists. The poor discriminative ability of models evidenced here is in contrast to good discriminative ability of models when applied to data collected by dermatologists, where models were previously shown to yield AUROC of 0.9451 (mucosal involvement model) and 0.9251 (lymphopenia model).^5^ This discrepancy in model performance may be due to difficulty in non‐dermatologists recognising important model variables including mucosal involvement, atypical target lesions, and Nikolsky sign, three of the four variables in the mucosal involvement model. Additionally, Nikolsky sign has the largest impact in both models' predictions.^4^ Training of non‐dermatologists in recognising these morphologies may improve model performance. As employed here, hospital dermatology triage systems with case and image review decrease unnecessary transfer of patients without SJS/TEN, especially given difficulty non‐dermatologists may have in consistently and accurately recognising model variables. In the original predictive model development, fever was noted more often in those with SJS/TEN than those with clinical mimickers.^4^ Here, fever was observed in 8/13 (61.5%) patients with SJS/TEN but only 3/34 (8.8%) patients without SJS/TEN. Thus, findings here support the inclusion of fever as an objective variable in future model development. As questionnaire results were used to guide transfer decision, a limitation of this study is that requesting centres may have answered questions to maximise probability of transfer acceptance. Future directions should focus on creating a predictive model identifying patients not only with SJS/TEN, but any patient with severe skin disease who would benefit from transfer to a hospital with inpatient dermatology.

## CONFLICT OF INTEREST STATEMENT

None to declare.

## AUTHOR CONTRIBUTIONS

**Hunter J. Pyle**: Data curation (equal); formal analysis (equal); writing—original draft (equal). **Arturo R. Dominguez**: Conceptualization (equal); investigation (equal); supervision (equal); writing—review & editing (equal). **Melissa M. Mauskar**: Conceptualization (equal); investigation (equal); supervision (equal); writing—review & editing (equal). **Cristina Thomas**: Conceptualization (equal); investigation (equal); supervision (equal); validation (equal); writing—review & editing (equal).

## ETHICS STATEMENT

This study was reviewed by our Institutional Review Board with exception as a quality improvement study.

## FUNDING INFORMATION

This article received no specific grant from any funding agency in the public, commercial, or not‐for‐profit sectors.

## Data Availability

The data that supports the findings of this study are available in the manuscript.
